# Supplementary material for: Are carbon emissions trading and green financial instruments synergistic? -Comprehensive quantitative research based on content analysis
Source: PLoS One. 2024 Mar 7;19(3):e0298601. doi: 10.1371/journal.pone.0298601 (PMC10919601; doi:10.1371/journal.pone.0298601)
Supplement: S1 Data — (ZIP) [file pone.0298601.s001.zip › Data/地级市Z_F工作报告.zip!/║■▒▒/╬Σ║║╩╨/└┤╫╘╣1⁄2╓┌║┼í╛┬φ┐╦╩2╛▌═°í┐_2018.docx]

各位代表：

现在，我代表市人民政府，向大会报告工作，请予审议，并请市政协各位委员和列席人员提出意见。

一、2017年工作回顾

刚刚过去的2017年，是极不平凡的一年。党的十九大胜利召开，确立了习近平新时代中国特色社会主义思想的指导地位，开启了全面建设社会主义现代化国家新征程。省第十一次党代会和市第十三次党代会胜利召开。武汉发展迈入新时代，踏上新征程，呈现新气象。

一年来，我们紧密团结在以习近平同志为核心的党中央周围，增强“四个意识”，坚定“四个自信”，把牢“四个着力”，在省委、省政府和市委的坚强领导下，聚力改革创新、奋力拼搏赶超，坚持做“行动政府”，全力推进中央重大战略贯彻落实，全力推进省委、省政府和市委部署安排精准落地见效，全市上下形成拼搏赶超强大气场，现代化、国际化、生态化大武汉建设迈出坚实步伐，圆满完成市十四届人大一次会议确定的目标任务，各项工作呈现出许多特色和亮点。

——复兴大武汉新征程豪迈起步。我们系统梳理新时代武汉发展新目标，进一步明确了武汉在全省、中部地区、长江流域、全国，乃至全球城市发展格局中的新定位，进一步明确了从目前到本世纪中叶实现中华民族伟大复兴的时间轴上复兴大武汉的时间节点、使命任务。武汉的全面复兴，在中华民族伟大复兴的洪流中滚滚向前！

——“三化”大武汉建设恢弘展开。我们高点推进长江空间主轴，依托沿线自然生态禀赋、城市历史文化积淀，打造历史之城、当代之城、未来之城；以整合统筹空港、陆港、水港为抓手，塑造国家中心城市功能品质；以东湖高新区、武汉开发区、临空港经开区、长江新城（新区）四大功能板块为牵引，构建多极支撑新格局。武汉，正向“中轴结构”发展，向“长江时代”跨越！

——创新发展气势强劲。我们创新实施系列发展举措，突破性发展新民营经济，大力推进招商引资“一号工程”、招才引智“一把手工程”，大力推进“四大资智聚汉工程”，大力推进四个国家新基地建设，大力推进“新两园”建设，大力推进“三办”改革，探索形成“大学+”“大湖+”新发展模式。海内外高端资本人才加速聚集，各种要素资源潜力加速释放。武汉，正站在新时代城市发展的风口！

——十件实事全面完成。我们坚持以人民为中心，大力推进一批惠民举措落地实施，解决了许多长期想解决而没有解决的难题，办成了许多过去想办而没有办成的大事。取消“九桥一隧一路”ETC车辆通行费，每年可为市民、企业节约10多亿元。武汉，正用民生温度彰显城市发展新高度！

——主要经济指标增速在全省实现由跟跑到领跑。我们强力扭转本轮周期性下滑态势，主要经济指标总量和增速在全省“双领先”。预计地区生产总值1.34万亿元左右，增长8%左右；全社会固定资产投资7800亿元以上，增长11%左右；社会消费品零售总额突破6000亿元，增长10%以上；地方一般公共预算收入1402.9亿元，增长11.2%；城乡居民人均可支配收入保持快速增长。蝉联全国文明城市、国家卫生城市荣誉称号，再次夺得“长安杯”，荣膺首批国家食品安全示范城市、中国旅游休闲示范城市，成功入选世界设计之都，再次获得C40城市奖，城市的认同感、美誉度、影响力显著提升。今天的武汉，正处在历史发展最好时期，正展现历史发展最好局面！

回顾过去一年，我们主要抓了以下工作：

（一）突出打好“工业翻身仗”，保持经济持续健康发展

工业经济强势回升。每周通报点评、每月协调督办，规模以上工业增加值增长8%左右，创36个月新高。新增千亿产业1个、百亿企业3户，东风本田成为首个千亿企业，新增规模以上工业企业204户。工业投资增长12%以上，技改投资是上年的2.5倍；武汉天马六代线、上汽通用二期等271个亿元以上工业项目建成投产。“中国制造2025”试点示范城市建设全面展开，获批国家信息光电子创新中心，首届中国工业设计展在汉举办。

生产性服务业提质增效。金融机构本外币存款余额24499.4亿元，增长10.4%；贷款余额23833.7亿元，增长14.8%，金融业增加值首次突破千亿元，居中部城市首位；物流业增加值1200亿元左右，增长8.9%左右；软件和信息服务业营业收入超过1800亿元，增长17%以上。

招商引资轰轰烈烈。校友招商、专场招商精彩纷呈，聘请54名招商大使、29名招才顾问、10名招才大使，各类招商活动签约金额2.58万亿元，实际到位资金8227亿元，创历史新高。实际利用外资96.5亿美元，居全国同类城市首位。扎实抓好项目落地，实施亿元以上项目开工专项行动，开工（开业）落地584个项目，总投资7030亿元，当年签约当年开工（开业）落地274个，涌现出光谷、车都、临空港速度。

区级经济整体跃升。三个国家级开发区拉动作用明显增强，规模以上工业增加值占全市45%。中心城区服务业加快发展，江岸、江汉、武昌区地区生产总值均超过千亿元。新城区工业经济加快壮大，规模以上工业增加值增长9.5%。“三乡工程”吸引社会资金投入160亿元，带动农民人均增收864元，成为推动农业农村现代化的新引擎。

（二）突出“四大资智聚汉工程”，加快培育发展新动能

资智聚汉掀起热潮。百万校友资智回汉热潮涌动，校友项目签约金额1.3万亿元，占招商引资总额50.4%。百万大学生留汉创业就业创历史新高，30.1万名大学毕业生留汉，新增落户大学生14.2万人。高校院所科技成果转化对接成果丰硕，在全国率先成立科技成果转化局，实现206项重大科技成果就地转化，总金额244亿元。海外科创人才来汉发展空前活跃，引进诺贝尔奖科学家4人、国家“千人计划”专家68人、海内外高层次人才392人。

创新要素加快汇聚。深入推进全面创新改革试验，一批经验做法在全国得到复制推广。创新平台功能增强，新增国家双创示范基地4个，居全国同类城市首位。创新企业快速发展，小米科技、科大讯飞等25家知名企业扎堆武汉，形成“第二总部”现象。创新成果持续涌现，发明专利申请量20603件、授权量7444件，分别增长13%、15%。全社会研发经费（R&D）投入强度稳步提升，达到3.2%。成立武汉知识产权审判庭，获批国家知识产权强市创建市。

新动能加速形成。四个国家新基地建设全面推进，国家存储器基地第一工厂提前封顶，国家航天产业基地火箭总装总调中心等项目开工，国家网络安全人才与创新基地在建项目总投资超过1000亿元，国家新能源和智能网联汽车基地吸引东风新能源、吉利汽车芯片等大项目落户。信息技术、生命健康、智能制造三大战略性新兴产业产值（营业收入）均增长17%以上，新认定高新技术企业1268家，高新技术产业产值净增千亿元，增长13%。新技术、新产业、新业态、新模式层出不穷。

（三）突出“三办”改革，加快破解发展难题

“三办”改革强力推进。深化“放管服”改革和法治政府建设，集成机构减窗口，集成流程减环节，集成信息提效率，全市“马上办”“网上办”“一次办”事项实现率分别为50%、45%、80%，市级平均每个事项办理时间压缩10.6天。向区级委托下放100项审批服务事项。审批“中介超市”上线运行。统计体制改革持续推进。商事制度改革不断深化，新增市场主体18.17万户，增长24.4%，市场主体总数首次突破百万户。

供给侧结构性改革持续深化。坚持去产能与优产能相结合，清理处置“僵尸企业”、空壳企业59户，出台“零土地”技改、“新两园”建设等政策，规模以上工业企业利润大幅增长。综合施策降成本，减轻企业负担241.6亿元。金融风险防控成效明显。控房价、防捂盘、稳市场，“六清理”行动扎实推进，住房租赁试点50条、大学毕业生八折租房购房政策引起强烈反响，房地产市场保持平稳健康发展。农业供给侧结构性改革扎实推进，农村集体产权制度改革、承包地“三权”分置改革进展顺利，农村产权交易市场辐射全省，交易规模26亿元，流转土地9.7万亩。

新民营经济突破性发展。率先出台政策措施，突出“四军三名”，掀起新民营经济发展热潮。招商引资民企到位资金4339亿元，民间投资愿望大幅回升，武汉民营经济焕发出勃勃生机。

（四）突出自贸区建设，不断增强内陆开放新优势

自贸区建设全面展开。湖北自贸区武汉片区挂牌运行。110项年度改革任务基本完成。率先推行科技成果所有权混合所有制改革，率先推出“科技悬赏奖”等创新举措。全国首个“海外留学回国人员创新创业板”在汉启动，首批30家高新技术企业集体挂牌。高端项目扎堆核心区，8公里地带吸引资本超过3000亿元，成为名副其实的“吸金区”。

国际通达能力持续增强。新开通武汉至悉尼、圣彼得堡等7条国际航线，首次开通洲际全货机航线,国际及地区旅客吞吐量继续保持中部第一。中欧（武汉）班列首次实现“三线出关”，率先开通“天天班列”，回程班列实载率居全国中欧班列之首。

口岸功能进一步完善。武汉新港空港综合保税区封关运营。武汉电子口岸·国际贸易“单一窗口”上线运行，快速通关模式初步形成。出口退税系统启动运行，可实现“秒退”。铁路汽车整车进口口岸获批，阳逻国际港进口肉类指定口岸投入使用，天河机场进境水果指定口岸获批运营。完成口岸货运量1200万吨，增长10%以上。

国际经济合作不断加强。对外承包工程及劳务合作营业额39.15亿美元，增长18%。服务外包合同签约额实现翻番，获评中国服务外包风采城市。中法生态示范城全面启动建设，一批法资企业和研发中心落户，总投资超过50亿元。

国际交流亮点纷呈。新增国际友好城市2个、友好交流城市6个，获批领事认证自办权。华创会、光博会、中法城市可持续发展论坛等大型国际活动成功举办。武汉国际马拉松、武汉国际赛马节、首届世界飞行者大会、首届武汉水上马拉松轮番上演，形成汉马、赛马、天马、水马“四马奔腾”之势，汉马举办两年好评如潮，跻身“中国马拉松大满贯”赛事。军运会筹办工作紧锣密鼓，会徽、吉祥物、口号正式发布。

（五）突出亮点区块建设，持续提升城市功能品质

亮点区块建设全面推进。确定长江新城（新区）规划选址，发布概念规划，严格管控起步区。成立长江新城管委会，创设区域股份合作制。国家级长江新区申报取得关键进展。发布长江主轴概念规划，交通轴、经济轴、生态轴、景观轴、文化轴“五轴一体”规划研究深入展开。左右岸大道示范段和沿江景观阳台开工建设。东湖城市生态绿心建设成效明显，东湖水质持续改善，东湖绿道二期建成开放，总长达102公里，成为江城靓丽风景线。

综合交通枢纽地位进一步强化。城建投资2361亿元，再创新高。天河机场T3航站楼建成投用，跻身全国最高等级机场行列。汉南通航机场正式启用。《武汉铁路枢纽总图规划》获批。阳逻国际港铁水联运工程建成投用。沌口长江大桥、武深高速武汉段、四环线西段、孝汉大道武汉段、机场快速路改线等实现通车。轨道交通建设战略性提速，开工5条线，首次实现一年开通3条线，1号线径河延伸线、8号线、21号线开通，全市通车总里程237公里，轨道交通占公共交通客运量比例达38%。

城市智能化管理深入推进。“易行江城”智慧交通管理系统不断完善，实现行车停车双诱导，高峰拥堵延时指数全国城市排名持续下降。智慧安防示范工程深入实施。智慧桥梁监测系统上线运行。手机支付广泛使用，加快迈向“移动生活城市”。城乡市容环境治理统筹推进，1913个行政村垃圾无害化处理率90%以上。

（六）突出“四水共治”，着力改善生态环境质量

系统治水成效显著。全面推进防洪水、排涝水、治污水、保供水，实施治水项目347个，投资230亿元，增长95%。全市防洪保障能力不断增强。16项重点排涝工程汛前投用，中心城区新增抽排能力490立方米/秒，比上年提升50%。城镇生活污水、工业园区废水、农业面源污染等治理全面加强，19条黑臭水体基本完成整治。561处“三无”老旧社区二次供水设施完成改造。农村饮水安全工程提档升级，受益人口近百万。国家海绵城市三年试点任务全面完成。

生态保护力度不断加大。落实推进共抓长江大保护实施意见，长江汉江岸线资源环境整治成效明显。绿色生态空间不断拓展，新改建各类公园81座，植树造林4.5万亩，建设绿地1.3万亩，戴家湖公园获中国人居环境范例奖。武钢飞灰填埋场、长山口生活垃圾预处理厂等项目建成投用，土壤污染防治有序推进，建筑弃土、危险废弃物等处置能力明显提升。空气质量优良天数255天，比上年增加18天。中央环保督察反馈问题当年整改任务全面完成。

生态文明体制进一步完善。领导干部自然资源资产离任审计全面展开。湖长制深化实施，河长制全面建立。长江汉江跨区断面水质考核奖惩及生态补偿全面启动。

（七）突出办好民生实事，切实增进民生福祉

公共财政用于民生领域支出1257.9亿元，增长14.4%。

精准扶贫深入推进。加大专项扶贫资金投入，重点推进产业扶贫、生态扶贫、健康扶贫，47528人脱贫销号、107个贫困村脱贫出列。

就业社保不断加强。新增就业19.84万人，扶持创业3.3万人。中心城区和新城区最低工资标准分别提高12.9%、13.6%。社会保险净增参保90.58万人次。全市统一的城乡居民基本医疗保险制度正式实施，城乡参保人员医保待遇总体提升。企业退休人员人均养老金、城乡居民基础养老金标准分别提高5.8%、20%。城乡低保、特困人员等困难群众救助供养标准综合提标31.5%，为历年最大增幅。新建老年宜居社区200个、社区老年人服务中心65家、农村老年人互助照料活动中心56家，老年证办理和高龄津贴发放实现网上帮办。建成保障房4.93万套，筹集大学毕业生租赁房8430套。

公共服务水平持续提升。新改扩建公益普惠性幼儿园34所、中小学校21所，分别新增学位7630个、19140个。城市公立医院综合改革全面推开，医疗机构药品加成全面取消，市、区属公立医院全部实现就诊“一卡通”、病历“一本通”、检查“一单通”。盘龙城国家考古遗址公园获批。琴台音乐节、斗鱼嘉年华、“知音号”等文化品牌效应不断彰显。市第十届运动会圆满举行。

社会治理创新扎实推进。安全生产形势总体平稳。平安武汉建设再上台阶，创设“网上群众工作部”，1872件信访积案结案化解100%。

国家安全、国防动员、军民融合、民防、消防、仲裁、保密、气象工作扎实推进，参事、文史、档案、民族、宗教、侨务、地方志工作不断加强，妇女、儿童、老龄、残疾人事业健康发展。

各位代表，看似寻常最奇崛，成如容易却艰辛。过去一年取得的成绩，得益于以习近平同志为核心的党中央的英明领导，得益于习近平新时代中国特色社会主义思想的指引，得益于省委、省政府和市委的正确领导，得益于全市上下的励精图治，得益于广大市民的大力支持。在此，我代表市人民政府，向全市人民，向给予政府工作大力支持的人大代表、政协委员，向各民主党派、人民团体和各界人士，向中央在汉单位和各类驻汉机构，向驻汉人民解放军、武警官兵，向参与城市建设的劳动者，向所有关心支持武汉发展的港澳台同胞、海外侨胞和国际友人，表示崇高敬意和衷心感谢！

我们也清醒地认识到，发展不平衡不充分依然存在，发展水平还不能满足市民日益增长的美好生活需要，主要表现为：发展质量效益还不够高，经济发展“稳”的基础还不牢固，传统产业支撑减弱，新动能成为主支撑还需时日；经济结构还不优，民营经济发展还不够；开放水平与先进城市和自身定位还有差距，国际化程度有待提高；城乡二元结构突出，城市功能品质仍需提升；民生社会事业发展水平还不够高，社会治理创新亟待加强；部分政府工作人员综合素质不能适应新要求，“四风”问题仍然存在。我们一定正视问题，切实推进解决。

二、2018年主要任务

2018年，是贯彻党的十九大精神的开局之年，是改革开放40周年，是决胜全面建成小康社会、实施“十三五”规划承上启下的关键一年，是武汉推动各项决策和工作全面落地见效的再落实之年，是武汉乘势而进、拼搏赶超的再奋进之年。我们要以习近平新时代中国特色社会主义思想为指导，深入贯彻党的十九大精神，按照省委、省政府和市委决策部署，坚持稳中求进工作总基调，坚持新发展理念，全面落实“七大战略”，推动高质量发展，坚决打好防范化解重大风险、精准脱贫、污染防治攻坚战，加快建设现代化、国际化、生态化大武汉，努力打造国家中心城市和世界亮点城市，为全面复兴大武汉不断夯实基础！

经济社会发展的主要预期目标是：地区生产总值增长7.8%左右；地方一般公共预算收入增长8%；全社会固定资产投资增长11%；社会消费品零售总额增长10.5%；城乡居民人均可支配收入增长与经济增长同步。全面完成省下达的节能减排任务。

重点抓好以下十个方面工作：

（一）扎实推进历史之城当代之城未来之城建设，优化城市空间战略布局

以长江文明之心、长江主轴、长江新城（新区）为重点，优化空间结构，重构城市格局，增强城市未来发展竞争力，托起新时代伟大城市梦想！

提升建设历史之城。在武昌古城、汉口历史风貌街区、汉阳归元片区，实施生态复修、老城复兴、文脉复归工程。规划建设新长江文明馆。推动文化交流、综合演艺、时尚创意、文化科技等功能区连片发展，建设世界级历史人文展示区。

优化建设当代之城。推进“五轴一体”，精心打造长江主轴。加快建设左右岸大道示范段和沿江景观阳台。提升景观照明，推进大桥美化，实施沿江、垂江道路综合改造。开展滨水岸线生态修复。

规划建设未来之城。全面做好规划、收储、管控、建设等工作。完成编制长江新城（新区）概念规划、总体规划和起步区城市设计。规划长江科学城、“城市水库”、综合管廊等项目。深化完善区域股份合作制，健全经社分开、统分结合的运行机制，积极引进央企、民企、外企等企业参与长江新城（新区）建设。争取获批国家级长江新区。

（二）扎实推进高效高新产业发展，加快构建现代化经济体系

坚持质量第一、效益优先，加快产业迈向价值链中高端，推动经济高质量发展。

巩固提升实体经济能级。加快发展先进制造业。围绕“一转型两手抓三融合”，深化“中国制造2025”试点示范城市建设，争创“中国制造2025”国家级示范区、国家军民融合创新示范区，加快建设国家信息光电子创新中心。实施“万千百工程”，加快打造万亿级光电子信息产业集群，推动6个千亿产业上水平，突出抓好20户百亿企业增长，加快推进一批准百亿企业成长壮大。抓好“两个50%”，做大做强11个支柱产业。规模以上工业增加值增长7.8%左右。落实好“零土地”技改政策，推进规模以上工业企业技改全覆盖，工业投资增长12%。

加快发展现代服务业。大力推进全国服务业综合改革试点城市建设，实施服务业六大跨越工程，打造九大特色鲜明、业态高端的服务业发展片区和商务功能区。加快建设中部金融中心，大力发展科技金融、普惠金融、绿色金融，引进金融机构8家以上，小微企业贷款增速、贷款户数、申贷获得率进入全国同类城市前列。推进全域旅游大发展，实施六大计划，擦亮“五色旅游”名片，打造“一轴一心一山三城百湖”新格局。加快发展现代物流和会展经济。推动汉正街等传统商圈转型发展。实施新消费引领计划，大力发展新零售等新业态。

加快发展现代农业。持续推进国家现代农业示范区建设，大力发展生产生活生态多功能融合、高附加值现代都市农业，促进一二三产业融合发展，实现农产品加工业产值2800亿元、乡村休闲旅游综合收入140亿元。开展“互联网+”现代农业行动，实施农村电商发展“百千万工程”。

深入推进招商引资、资智聚汉。紧紧围绕“八个延伸”，持续推进招商引资“一号工程”和招才引智“一把手工程”，突出国际招商、海外招商，实际利用外资增长13%。持续推进“四大资智聚汉工程”，加快发展校友经济、菁英经济、院士经济、“海归”经济等新经济。深化百万大学生留汉创业就业工程，落实好大学毕业生落户、住房、收入系列政策，综合提升各类双创平台功能，努力打造“大学生最友好城市”，激励大学生用奋斗创造美好生活。深化百万校友资智回汉工程，推动新一轮校友招商。深化高校院所科技成果转化对接工程，组织5场大型对接活动，大幅提升在汉高校院所科技成果就地转化率。深化海外科创人才来汉发展工程，探索建设国际人才自由港，规划建设海外创新中心，继续引进诺贝尔奖科学家，举办国际创客大赛，聚集全球创新能量。创新推进智库建设。我们要深化“大学+”模式，优化经济结构，大力发展新时代校友经济，助校友成就事业，为城市赢得未来！

进一步做强区级经济支撑。推动国家级开发区改革和创新发展，开展二次创业，打造“五谷”“四都”“三港”，进一步增强极化效应。加快产业优化升级，全面推进“新两园”建设，各中心城区分别建设2个以上小微企业科创园，国家级开发区、跨三环线中心城区和新城区分别建设1个以上现代产业园，提高园区投入产出比。支持中心城区加快推动服务业迈向中高端，支持新城区加快推进新型工业化。开展“项目落地建设竞赛年”活动，统筹推进210个市级重大项目建设，确保京东方10.5代线、东风新能源示范园等重大项目开工建设，力争往年开工项目投产2/3以上、往年签约项目开工2/3以上、当年新签约项目开工1/3以上，切实推进已签约项目加快落地见效。

（三）扎实推进创新型城市建设，加快创新引领发展

扩大科教资源优势，优化创新生态环境，最大限度激发创新活力，最大限度释放创新动能。

完成全面创新改革三年试验任务。提升全面创新改革试验水平，高质量完成25项国家授权先行先试任务，积极申报全国科技保险示范区，加快推广全国创新改革经验，打造全球创新网络重要节点城市。

建设高水平创新平台。支持优化现有科技创新基地，加快建设武汉光电国家研究中心，争取建设国家实验室，支持打造大科学装置、国家研究中心等重大创新平台，积极申报综合性国家科学中心。支持在汉重点高校“双一流”建设，支持“三超”光纤传输、5G商用、量子通信、IPv6、人工智能、大纤维等领域重大产业项目科技攻关，抢占前沿技术产业化先机。

开展创新主体成长专项行动。建立健全高新技术企业梯次成长机制，培育瞪羚企业500家、科技“小巨人”企业1000家，新增高新技术企业500家。引进知名企业研发机构30家。积极创建国家知识产权强市，打造中部知识产权运营中心，发明专利申请量、授权量均增长15%。全社会研发经费（R&D）增长10%，高新技术产业产值、战略性新兴产业产值均增长15%以上。

加快建设四个国家新基地。以基地建设为牵引为依托，加快战略性新兴产业布局。加快建设国家存储器基地，确保第一工厂投产、力争量产，推进第二、第三工厂和配套产业项目建设，组建国家先进存储产业创新中心、存储芯片联盟。加快建设国家航天产业基地，推进火箭及运载服务、材料、低轨卫星和应用等领域重大项目。加快建设国家网络安全人才与创新基地，引进大数据、云计算、物联网等新一代信息技术领域高端人才和优质项目，打造特色数字经济体系。加快建设国家新能源和智能网联汽车基地，紧盯汽车轻量化、电动化、智能化、网联化趋势，大力发展下一代汽车，探索智能汽车、智慧交通融合发展新模式。

（四）扎实推进重要领域改革攻坚，不断塑造制度新优势

更高起点谋划，更大力度突破，变试点为示范、变试验为经验，让改革成果加速转化为发展成果！

深化供给侧结构性改革。完善产业结构调整负面清单，深化“三去一降一补”，在“破”“立”“降”上下功夫。支持宝武集团、神龙公司等企业创新发展。实施质量提升三大工程，争创国家标准化综合改革试点城市、全国质量强市示范城市。积极防范金融风险，深化投融资体制改革。实施上市公司倍增三年行动计划，直接融资增长10%。落实降本增效政策，降低企业成本260亿元。加快建立多主体供给、多渠道保障、租购并举的住房制度，推进集体建设用地建设租赁住房试点、培育和发展住房租赁市场试点。加大住房公积金对大学毕业生等群体租房支持力度，加快建设大学毕业生保障性住房，高标准建设青年城、青年苑，筹集租赁房60万平方米。加快分类改革，“一企一策”推进市属国企重组整合，深化国资国企混合所有制改革。开展第四次全国经济普查。

深化新民营经济发展。实施中小企业培育、管理能力提升、融资服务优化、民营企业家成长四大工程，培育名企、名品、名家。聚焦“四新”经济，分类制定实施专项扶持政策，建设一批新民营经济创新发展区。激发和保护企业家精神，引导支持新近毕业大学生、“海归”人才、科研院校技术人员、职业经理人等投身新民营经济发展。加快新兴领域知识产权保护制度建设，切实保障民营经济主体合法权益，加快打造新民营经济聚集地。

深化“放管服”改革。推进政府职能转变，加强市、区综合行政审批联动改革。推进“一次办”向“马上办”、“马上办”向“网上办”改革，加快实现“网上办为常态、网下办为例外”，扩大“不用办”领域。深化商事制度改革，推进“证照分离”“一址多照”、市场监管“八双”机制改革试点，启动创建国家网络市场监管与服务示范区。完善事中事后监管。

深化生态文明体制改革。深入开展领导干部自然资源资产离任审计。全面完成国家水生态文明城市试点任务。实施长江汉江跨区断面水质考核奖惩及生态补偿机制。创造性开展河长制、湖长制工作，完善森林林地、湿地、城市绿地保护制度。落实碳排放达峰行动计划，开展气候适应型城市建设试点。加快推行排污许可制。持续推进中央环保督察反馈问题整改。

（五）扎实推进开放型经济发展，持续提升开放质量水平

承接“一带一路”，挺起长江经济带脊梁，更高水平引进来，更大力度走出去，加快打造内陆开放新高地。

强化自贸区引领示范。坚持“双自联动”，重点推进70项改革，打造新兴产业发展区、全面改革试验田、内陆开放新窗口。鼓励外企设立外资研发中心，新增10家跨国公司研发机构。开展知识产权综合管理改革试点，推进投贷联动、知识产权质押融资等方面创新。积极申报建设跨境电商综合试验区。探索建设自由贸易港。

深度融入“一带一路”国际合作。加快建设中法生态示范城，打造开放合作新标杆。支持企业拓展沿线国家和地区市场，带动产品出口和国际产能合作；大力开展工程承包和境外投资，带动优质产品、优势装备和技术标准输出。鼓励企业开展跨国并购，支持企业提高国际化经营能力。深化汉口北市场采购贸易试点。推进服务贸易创新发展试点，积极发展服务外包产业。

拓展国际大通道。加快推进国际陆运枢纽、国际航空枢纽、国家多式联运枢纽和武汉长江中游航运中心建设。新开通国际及地区航线3条，优化提升中欧（武汉）班列运营能力，扩大班列辐射范围。提升阳逻国际港功能，加快建设航运产业总部区，支持“江海直达”新型船舶建造，完善近洋航线网络，申报国家汽车整车进口水运口岸，建成武汉多式联运海关监管中心。推进汉南港区一类水运口岸建设。

进一步扩大对外交往。积极争取重大国际性活动、国际组织、国际机构落户武汉，加强国际友城工作。推进国际社区、国际医院、国际学校建设。办好武汉国际马拉松、武汉水上马拉松、武汉网球公开赛、武汉国际时装周等国际节会赛事活动。高标准做好军运会筹办工作，征集创作军运会会歌和主题推广曲。

深化长江中游四省会城市合作。推动武汉产业链、公共服务资源向周边城市延伸，拓展“飞地经济”，探索武汉大都市区发展。

（六）扎实推进城建跨越，着力增强城市综合承载能力

坚持最优方案、最大力度、最小动静、最佳效果，高标准建设管理，提升国家中心城市建设新水平。

加快空港陆港水港整合统筹。完成城市总体规划修编，启动土地利用总体规划修编全国试点。强化综合交通枢纽功能。加快实施《武汉铁路枢纽总图规划》。持续推动高铁进机场，规划天河北站，围绕“高铁+城铁+地铁+机场”目标，打造功能互补、相得益彰的复合交通体系。加快推进武西客专直通线、新汉阳火车站、光谷火车站等项目。建成江北快速路，开工建设阳逻国际港铁水联运二期，加快推进江北铁路、香炉山货场等项目。启动武汉大都市区交通基础设施规划前期研究，加快建设武阳（新）高速、武大（悟）高速等项目。

以地铁引领公共交通。开通7号线、27号线，开工2条线路，续建11条线路。完善市民公共出行体系，推动常规公交、共享单车、共享汽车、公交微循环等交通方式与地铁无缝衔接，让低碳绿色成为生活时尚。加快完善“五环二十四射”快速路网，建成三阳路过江通道、四环线南段等项目，力争开工建设两湖隧道。打好补齐南湖、白沙洲地区生态环境和综合交通短板大会战。建成微循环道路80条。

提升城市智能化精细化管理水平。做好军运会环境整治综合提升工作。延伸拓展“易行江城”智慧交通管理系统，推动各类智慧管理平台深度整合、有机融合。实施城管精细化提标行动，精准治理交通拥堵、安全隐患、违法建筑、街巷环境卫生等问题。综合整治老旧社区“三线一网”，持续改善老旧社区基础设施。加强“三站一场”秩序管理。建立城市有机更新机制，积极推进“三旧”改造，按照“建设快、动作小、投资省、作用大”原则，加强骨干路网等城市基础设施微改造。全面开展生活垃圾分类试点。持续推进“厕所革命”。

（七）扎实推进美丽武汉建设，努力提供更多优质生态产品

坚持生态优先、绿色发展，加快建设滨水生态绿城，让市民在家门口畅享高品质生活。

持续推进“四水共治”。坚持把修复长江生态环境摆在压倒性位置，实施共抓长江大保护“五大行动”。推进“大湖+”主题功能区建设。科学防洪水，强化两江干堤、连江支堤、中小河流、水库山洪、湖泊民垸等防线，重点提升主要连江支流防洪能力。系统排涝水，深入推进海绵城市建设，新改扩建城排泵站12座，中心城区抽排能力新增400立方米/秒。强力治污水，推进老旧城区雨污分流改造，实施工业污染源全面达标排放计划，强化东湖、南湖、黄孝河、机场河等水体综合整治。全力保供水，完成堤角、白鹤嘴等水厂扩建改造，分区域开展直饮水试点，积极开展中水回用试点，继续推进老旧社区二次供水设施改造，加强应急水源地建设。

深入开展大气土壤污染治理。实施拥抱蓝天行动年度方案，持续增加空气质量优良天数。巩固燃煤锅炉整治成果，推进燃煤炉窑综合整治。投放新能源公交车560辆。提升液化天然气储备能力。强化道路和建筑工地扬尘、移动源排放等污染管控。制定土壤污染治理与修复规划，滚动实施年度防治项目。加快北洋桥生活垃圾填埋场生态修复。积极推进北湖生态文明试验区建设。

大力实施全域增绿提质。以提升文化内涵为重点，高水平建设东湖绿道三期，加强环境整治、服务配套和运营管理，全面实施“景中村”改造，优化并严控环湖天际线，打造世界名湖、生态典范、城市绿心、最好景区。积极创建国家生态园林城市。开展迎军运园林绿化提升行动，完成“三基本、四覆盖”任务。大力推进通道绿化、生态绿楔、毁损山体补绿、郊野公园项目，加快建设口袋公园、“三小”绿地、控规绿地，提升“绿容率”，让武汉处处见绿、层层彩化、时时花香。

（八）扎实推进“三乡工程”，加快实现乡村振兴

重塑城乡关系，加快城乡融合，推动实现“三个率先”，努力让农村与城市发展同步、农民与市民机会均等。

实施“三乡工程”拓面提质三年行动计划。整体规划、完善配套、全域推进。推动市民下乡，支持市民租用农村空闲农房和农地资源，增加农民财产性收入。推动能人回乡，出台支持政策，激励能人返乡创业，带动农民就业。推动企业兴乡，引导工商企业到农村投资兴业，开展整村股份合作开发。新城区启动创建都市田园综合体，建设一批特色村镇、共享农庄。持续推进农村基础设施建设。

深化农业供给侧结构性改革。实施质量兴农战略。完善承包地“三权”分置制度，完善农民闲置宅基地和闲置农房政策，深入推进农村集体产权制度改革。实施培育新型农业经营主体工程，积极发展多种形式适度规模经营，扶持各类新型主体，更好带动小农户和现代农业发展有机衔接。实施农业科技提升工程，加强农村专业人才队伍建设。深化供销社综合改革。

坚决打赢脱贫攻坚战。推进“五个一批”“六大工程”，深化“百企帮百村”行动，确保已建档立卡的贫困人口脱贫销号、所有贫困村脱贫出列，持续巩固脱贫成果，做到扶真贫、真扶贫，脱真贫、真脱贫。全面建成小康路上，一个都不能少！

实施乡村治理提升行动。健全完善自治、法治、德治相结合的乡村治理体系，拓展网格化服务管理，建设平安村湾。培育文明乡风、良好家风、淳朴民风，不断提高乡村社会文明程度。持续改善农村人居环境，全面建成投用乡镇生活污水处理设施，完善配套污水收集管网建设，开展村庄生活污水治理试点。推进农业面源污染防治，实现禁养区全面退养。实施公共服务水平提升工程，加快优质教育、文化、医疗等公共服务向农村延伸。

（九）扎实推进文化强市建设，持续提升文化软实力

传承历史文脉，弘扬现代文明，铸城市之魂，扬城市之韵，建设文化小康！

繁荣文化事业。完善公共文化服务体系，深入实施文化惠民工程，新建改造一批群众性运动休闲场所设施，加强政府购买公共文化服务。在亮点区块规划建设公共文化设施，推进武汉广电中心、武汉中心书城建设，规划建设校友文化主题公园。打造琴台中央文化艺术区，加快建设琴台美术馆，办好琴台音乐节。侯卫东官场笔记举办国际杂技艺术节、国际渡江节、“武汉之夏”等文体活动。建成开放盘龙城遗址博物院、武汉自然历史博物馆。持续推进万里茶道申报世界文化遗产，举办大河对话国际论坛。推动京剧、汉剧、楚剧传承发展，振兴武汉“戏码头”，争取获批成立全国戏曲演出交流中心。推动武汉文化走出去，开展城市形象全球营销。加强文化艺术人才队伍建设。完成国家文化消费试点城市工作任务。

发展文化产业。出台文化创意产业创新发展政策，加快推动文化产业成为支柱产业。以成功入选世界设计之都为契机，做大做强工程设计、工业设计，加快培育发展时尚设计、动漫游戏、电子竞技、网络文化、电子出版、艺术品交易等文化创意产业。拍摄校友资智回汉主题影视剧，策划打造大型实景演出，举办直播节、电商节等新型节会。争创国家文化产业创新试验区。

建设文明城市。培育和践行社会主义核心价值观，深化中国特色社会主义和中国梦宣传教育。开展“高水平建设全国文明城市”主题活动，实施公民道德建设工程，扎实开展志愿服务、诚信建设等活动，持续抓好文明村镇、文明单位、文明校园、文明家庭创建工作。

（十）扎实推进保障和改善民生，更好满足市民美好生活需要

悠悠万事，民生为大。我们要坚持以人民为中心，兜底线、保基本、提品质，让市民获得感更充实、幸福感更真实、安全感更踏实！

实施城乡居民增收激励计划。推进更高质量和更充分就业，大力开发就业岗位，城镇新增就业19万人。大规模开展职业技能培训，提升城乡贫困家庭子女、农民工等就业技能。拓宽居民劳动收入、财产性收入渠道，努力扩大中等收入群体，增加低收入者收入。深化工资收入分配制度改革，完善职工工资正常增长机制，推动落实大学毕业生指导性最低年薪标准。健全三方协调机制，构建和谐劳动关系，创建市级模范和谐企业40家。

完善多层次社会保障体系。实施全民参保计划，社保扩面新增40万人次，提高城镇职工、城乡居民基本养老保险待遇水平，全面实施统一的城乡居民基本医疗保险、大病保险和医疗救助制度。扩大政府购买社会救助服务试点范围。实施棚户区改造5.05万户，建成保障房3万套，发放租赁补贴6000户。全面实施“互联网+居家养老”行动计划，支持兴办各类医养结合养老服务机构，大力发展社区为老服务，创建300个老年宜居社区。对1万户残疾人家庭居家环境实施无障碍改造。

扩大优质教育供给。建设国家学前教育改革发展实验区，全面实施幼儿园配建政策，加快已配建幼儿园移交，切实加强幼儿园安全规范管理，让家长和社会放心。深化义务教育优质均衡发展体制机制改革，开展普通高中“领航学校”、特色学校建设,大力推进校园足球试点学校建设。加快发展现代职业教育和特殊教育,进一步加强市属高校建设。实施教育国际化三年行动计划。加强师德师风建设，培育高素质教师队伍。

推进健康武汉建设。深化城市公立医院综合改革，严控医疗费用不合理增长，严控药品、耗材占比。有序推进基层医疗卫生综合改革，积极构建紧密型医联体，增加基层用药范围和种类，促进基层首诊。创建区域性国家儿童医疗中心，探索儿科医生定向培养，增加儿科科室，开工建设武汉儿童医院妇幼综合大楼，加快部分区妇幼保健医疗机构建设。大力发展高端精准医疗。深入推进计划生育转型发展。

打造共建共治共享社会治理格局。推进“红色引擎工程”，以基层党建引领基层社会治理创新，推进治理资源向社区下沉。充分发挥社会组织作用。组织居委会、村委会依法换届。建设更高水平的平安武汉，深入开展“万名警察进社区”活动，完善立体化社会治安防控体系，创建“五无”平安社区。开展安全生产打非治违专项行动，坚决防范遏制重特大事故发生。巩固提升国家食品安全示范城市建设水平。加强社会信用体系建设。深入实施“七五”普法。扎实开展双拥共建。完善多元化纠纷解决机制。充分发挥“网上群众工作部”作用，积极化解信访积案。

今年，我们将继续办好十件实事。

1、新增公共停车位1.5万个、人防地下停车位1.3万个，缓解市民停车难。

2、新改扩建公益普惠性幼儿园40所、中小学校20所，分别新增学位1万个、2.2万个，服务“二孩”政策和大学生留汉创业就业。

3、新改扩建社区老年人服务中心50家，提供助餐、助洁、助医、远程照护等居家养老服务。

4、每个中心城区在3-5个老旧小区多层住宅开展加装电梯试点，鼓励新城区开展试点。

5、新改扩建城区公共厕所100座、旅游厕所100座，改造农村户厕1.8万户。

6、绿化高架立柱1万余个，建设林荫道50条、街心公园40个，提档升级道路绿化景观510公里。

7、为12万名适龄妇女进行免费“两癌”筛查，新改扩建3家区级妇幼保健医疗机构，全市二级以上公立综合医院全部开设儿科独立病区，增加儿科病床600张，三级公立医院儿科24小时提供急诊服务，新增儿科医生150名。

8、为1900个老旧小区提供基本物业服务，推进“红色物业”拓面提质，实现老旧小区物业管理全覆盖。

9、严控工地噪音污染，投诉处置率100%；在主干路、环线、快速路等城市高架上，新装、加装、改装隔音屏50处。

10、组织专业院团、大学生进社区（村）免费文艺演出1000场，开展“家庭教育公益大讲堂”进社区（村）活动2000场。

各位代表！

新时代、新目标，政府要有新气象，更要有新作为。我们要全面增强“八种本领”，大力弘扬为民奉献、拼搏赶超、改革创新、落地见效精神，来真的、干实的、不搞虚的，建设人民满意的服务型政府。

加强政治建设，增强“四个意识”。坚决维护以习近平同志为核心的党中央权威和集中统一领导，坚决贯彻中央方针政策，坚决落实省委、省政府和市委部署要求。严格执行新形势下党内政治生活若干准则，完善和落实政府议事规则和决策程序。精心组织开展“不忘初心、牢记使命”主题教育，坚持“两学一做”学习教育常态化制度化。

加强法治建设，推进依法行政。认真落实法治政府建设实施方案。深化行政执法体制改革，全面启动行政复议体制改革，大力推进行政机关负责人出庭应诉。主动接受人大法律监督、工作监督和政协民主监督，强化审计监督，畅通社会监督渠道。支持监察体制改革。

加强廉政建设，恪守公仆本色。认真落实全面从严治党主体责任和“一岗双责”，深入推进政府系统党风廉政建设和反腐败工作。健全工程建设、土地出让、政府采购、产权交易等重点领域腐败风险防控机制，坚决查处损害群众利益的问题。

加强作风建设，强化落实能力。不驰于空想，不骛于虚声，务实重行，真抓实干。严格落实中央八项规定实施细则以及省、市实施办法，开展正风除弊行动，进一步纠正“四风”和“新衙门作风”，按照“抓落实、优服务、惩蝇贪”要求，做到“五个坚持”，重塑为民办事体制机制。严格绩效管理，强化“三层督办”，加大问效问责。大兴调查研究之风，建立健全大数据支撑科学决策机制，对市民最盼、最急、最忧、最怨的问题，抓住不放，认真解决，收到实效，以造福人民为最大政绩，让市民对政府工作过程和结果双满意，用政府新作为，展示城市新形象！

各位代表！征程万里风正劲，重任千钧再扬鞭。让我们更加紧密地团结在以习近平同志为核心的党中央周围，在省委、省政府和市委的坚强领导下，不忘初心、牢记使命，以时不我待、只争朝夕的精神投入工作，全心全意为老百姓谋幸福，尽力尽责为大武汉谋复兴，在新时代伟大征程上，奋力谱写武汉发展壮丽篇章！
